# Supplementary material for: Unmet need for treatment-seeking from public health facilities in India: An analysis of sociodemographic, regional and disease-wise variations
Source: PLOS Glob Public Health. 2022 Apr 19;2(4):e0000148. doi: 10.1371/journal.pgph.0000148 (PMC10022036; doi:10.1371/journal.pgph.0000148)
Supplement: S1 Table — (DOCX) [file pgph.0000148.s001.docx]

**S1 Table. Disease classification and coding of the NSS data used in the analysis.**

| SL  NO | Disease condition/s | Reported diagnosis in NSS |
| --- | --- | --- |
| 1 | Infections | Fever with loss of consciousness or altered consciousness, Malaria Fever due to Diphtheria, Whooping Cough , All other fevers (Includes typhoid, Fever with rash/ eruptive lesions and fevers of unknown origin, all specific fevers that do not have a confirmed diagnosis) , Tuberculosis, Filariasis, Tetanus, HIV/AIDS, Other sexually transmitted diseases, Jaundice, Diarrheas/ dysentery/ increased frequency of stools with or without blood and mucus in stools and Worms infestation |
| 2 | Cancers | Cancers (known or suspected by a physician) and occurrence of any growing painless lump in the body |
| 3 | CVDs | Hypertension and Heart disease: Chest pain, breathlessness |
| 4 | Respiratory | Acute upper respiratory infections (cold, runny nose, sore throat with cough, allergic colds included), Cough with sputum with or without fever and NOT diagnosed as TB and Bronchial asthma/ recurrent episode of wheezing and breathlessness with or without cough over long periods or known asthma) |
| 5 | Gastro–intestinal | Diseases of mouth/teeth/gums, Pain in abdomen: Gastric and peptic, ulcers/ acid reflux/ acute abdomen, Lump or fluid in abdomen or scrotum and Gastrointestinal bleeding |
| 6 | Blood Disorders | Anaemia (any cause), Bleeding disorders |
| 7 | Endocrine | DIABETES, Under-nutrition Goitre and other diseases of the thyroid and Others (including obesity) |
| 8 | Psychiatric | Mental retardation, Mental disorders, Headache, Seizures or known epilepsy, Weakness in limb muscles and difficulty in movements, Stroke/ hemiplegia/ sudden onset weakness or loss of speech in half of body and Others including memory loss, confusion |
| 9 | Injuries | Accidental injury, road traffic accidents and falls, Accidental drowning and submersion, Burns and corrosions, Poisoning, Intentional self-harm, Assault and Contact with venomous/harm-causing animals and plants |
| 10 | Eye | Discomfort/pain in the eye with redness or swellings/ boil, Cataract, GLAUCOMA, decreased vision (chronic) NOT including where decreased vision is corrected with glasses, Others (including disorders of eye movements – strabismus, nystagmus, ptosis and adnexa). |
| 11 | Ear | Earache with discharge/bleeding from ear/ infections and Decreased hearing or loss of hearing |
| 12 | Skin | Skin infection (boil, abscess, itching) and other skin disease |
| 13 | Musco-skeletal | Joint or bone disease/ pain or swelling in any of the joints, or swelling or pus from the bones and Back or body aches |
| 14 | Genito–urinary | Any difficulty or abnormality in urination and pain the pelvic region/reproductive tract infection/ Pain in male genital area, Change/irregularity in menstrual cycle or excessive bleeding/pain during menstruation and any other gynaecological and andrological disorders incl. male/female infertility |
| 15 | Obstetrics | Pregnancy with complications before or during labour (abortion, ectopic pregnancy, hypertension, complications during labour), Complications in mother after birth of child and Illness in the new born/sick new born |
| 16 | Others | Symptom not fitting into any of above categories and could not even state the main symptom |
| Note: Based on the ICD-10 classification World Health Organization. (2010). International Classification of Diseases-ICD. | | |
