# Supplementary material for: Unmet need for treatment-seeking from public health facilities in India: An analysis of sociodemographic, regional and disease-wise variations
Source: PLOS Glob Public Health. 2022 Apr 19;2(4):e0000148. doi: 10.1371/journal.pgph.0000148 (PMC10022036; doi:10.1371/journal.pgph.0000148)
Supplement: S2 Table — (DOCX) [file pgph.0000148.s002.docx]

**Table S2.** **Description and Scale of measurements of explanatory variables**

| **S. No** | **Variable Level** | **Description and Coding** | **Scale of measurements** |
| --- | --- | --- | --- |
| 1 | Age of patients | Age of the patients in completed years and it is directly associated with the life style of the persons and groups in four category 0-14, 15-35, 36-59 and 60 and above Years. | Ordinal |
| 2 | Gender of the patients | Gender of the patients categorised as male and female which many of the studies highlighted the gender differentials in using treatment seeking behaviour. | Nominal |
| 3 | Education of the youth | Educational status of the patients was grouped into Illiterate, Up to Primary, Middle and Secondary and above education. | Ordinal |
| 4 | Marital Status of the patients | Marital status of the patients also bearing on life style. Marital status is reported as the time of the survey and categorised into never married, currently married and others (widowed/divorced/separated etc.) | Nominal |
| 5 | Relation to household’s head | Patients’ relation with the household’s head grouped into Self, Spouse of head, Unmarried child, married child, Spouse of child and Others. | Nominal |
| 6 | Religion of the households | Religious affiliation is another’s a contextual factor which have bearing on life styles of the patient’s religious brief/stigma and grouped into Hindu, Muslim and Others. | Nominal |
| 7 | Social group | For each household surveyed, the social group to which the head of the household belonged to was recorded irrespective of the actual social group to which the individual members belonged. In NSS data caste categories are classified as Scheduled Caste (SC), Scheduled Tribe (ST), Other backward class (OBC) and Others. In our study we merged first two categories, SC and ST to one category as SC/ST. Caste/tribe has significant relevance in the Indian context for any social and health indicator. Under the *Varna* system, humans were divided into four classes. The bottommost rung of this system includes 'scheduled castes/tribes,' who were allowed to do derogatory and scavenging work only. People from these strata lack access to information and health services while the 'other backward classes’ (OBC) comprise those deprived of health services primarily due to social and economic constraints. The 'other' category comprises those not comprised in any of the above categories | Nominal |
| 8 | Economic status of the households | It is the best on the MPCE (Monthly Per Capita Consumption Expenditure). Total households divided into five classes (Poorest, Poorer, Middle, Richer and Richest) of MPCE, where the Poorest means the level of MPCE below which 20 per cent of the households lie, Poorer class, the level below which 40 per cent of the households lie, and so on. | Ordinal |
| 9 | Place of residence | This variable is derived from the question sector of the residence. It is dichotomous variables (Rural and urban) according the where the patients were living at the time of the survey. | Nominal |
| 10 | Region of residence | To examine the regional variation of treatment seeking behaviour, India was divided into six regions based on geographical location and cultural settings. The six regions consist of North (Jammu and Kashmir, Himachal Pradesh, Punjab, Haryana, Rajasthan, Delhi and Uttaranchal), Central (Uttar Pradesh, Madhya Pradesh and Chhattisgarh), East (Bihar, Jharkhand, West Bengal and Orissa), North-East (Arunachal Pradesh, Assam, Manipur, Meghalaya, Mizoram, Nagaland and Tripura), West (Gujarat, Maharashtra and Goa), and South (Andhra Pradesh, Karnataka, Kerala and Tamil Nadu). | Nominal |
