# Supplementary material for: Unmet need for treatment-seeking from public health facilities in India: An analysis of sociodemographic, regional and disease-wise variations
Source: PLOS Glob Public Health. 2022 Apr 19;2(4):e0000148. doi: 10.1371/journal.pgph.0000148 (PMC10022036; doi:10.1371/journal.pgph.0000148)
Supplement: S3 Table — (DOCX) [file pgph.0000148.s003.docx]

**Table S3. State-wise unmet need for treatment seeking from any sources and unmet need for treatment seeking from any public health facilities among those who have suffered/suffering from any disease in India, NSS, 2004-2018**

| **States/Union Territory** | **unmet need for treatment seeking from any sources (%)** | | | **unmet need for treatment seeking from any public health facilities (%)** | | |
| --- | --- | --- | --- | --- | --- | --- |
|  | **NSS-2004** | **NSS-2014** | **NSS-2018** | **NSS 2004** | **NSS-2014** | **NSS-2018** |
| Andaman and Nicobar Islands | 2.5 | 10.6 | 2.4 | 11.8 | 21.5 | 9.3 |
| Andhra Pradesh | 14.2 | 10.3 | 1.7 | 60.2 | 75.0 | 70.0 |
| Arunachal Pradesh | 2.8 | 20.6 | 5.9 | 11.4 | 0.8 | 8.0 |
| Assam | 13.9 | 26.6 | 4.6 | 39.1 | 15.7 | 40.0 |
| Bihar | 12.0 | 35.7 | 10.2 | 63.7 | 53.2 | 51.2 |
| Chandigarh | 4.4 | 0.0 | 0.4 | 49.5 | 59.5 | 51.1 |
| Chhattisgarh | 8.6 | 25.5 | 2.7 | 66.4 | 52.6 | 49.1 |
| Dadra and Nagar Haveli | 6.2 | 28.3 | 0.0 | 85.8 | 30.0 | 42.4 |
| Daman and Diu | 3.5 | 4.8 | 0.0 | 89.2 | 91.8 | 72.8 |
| Goa | 3.9 | 1.2 | 0.2 | 53.9 | 70.3 | 41.0 |
| Gujarat | 8.7 | 6.2 | 1.4 | 68.6 | 62.2 | 68.0 |
| Haryana | 2.8 | 4.5 | 1.7 | 79.2 | 84.7 | 76.6 |
| Himachal Pradesh | 1.5 | 5.3 | 2.6 | 28.2 | 49.7 | 28.4 |
| Jammu and Kashmir | 8.7 | 7.8 | 0.7 | 38.6 | 48.4 | 27.0 |
| Jharkhand | 0.8 | 29.7 | 8.9 | 60.8 | 47.4 | 56.6 |
| Karnataka | 13.2 | 5.9 | 2.5 | 56.3 | 72.5 | 67.4 |
| Kerala | 9.3 | 4.8 | 0.5 | 54.8 | 60.4 | 49.8 |
| Lakshadweep | 6.8 | 2.0 | 0.0 | 5.7 | 23.4 | 18.5 |
| Madhya Pradesh | 7.4 | 8.3 | 2.4 | 59.8 | 63.7 | 60.3 |
| Maharashtra | 7.8 | 5.7 | 2.1 | 71.6 | 74.4 | 68.1 |
| Manipur | 1.5 | 21.6 | 0.3 | 20.7 | 38.0 | 12.2 |
| Meghalaya | 39.5 | 41.1 | 0.0 | 20.4 | 17.5 | 38.3 |
| Mizoram | 15.0 | 28.6 | 11.6 | 5.7 | 27.3 | 19.5 |
| Nagaland | 0.0 | 80.0 | 27.3 | 24.6 | 3.9 | 17.4 |
| Delhi | 4.6 | 11.3 | 2.1 | 72.7 | 70.7 | 50.7 |
| Puducherry | 24.5 | 10.5 | 0.7 | 29.9 | 55.2 | 33.7 |
| Punjab | 3.0 | 8.1 | 2.5 | 71.3 | 74.4 | 80.6 |
| Rajasthan | 7.1 | 8.6 | 2.6 | 46.1 | 54.2 | 51.4 |
| Sikkim | 26.3 | 24.1 | 4.9 | 5.2 | 14.5 | 39.3 |
| Tamil Nadu | 15.5 | 6.6 | 0.6 | 57.7 | 59.4 | 43.7 |
| Telangana | 15.5 | 6.3 | 0.8 | 57.7 | 79.6 | 76.0 |
| Tripura | 5.3 | 19.2 | 7.5 | 34.1 | 35.3 | 58.2 |
| Uttar Pradesh | 16.6 | 14.3 | 2.8 | 62.2 | 70.4 | 74.2 |
| Uttarakhand | 7.6 | 13.1 | 2.0 | 66.5 | 44.9 | 52.5 |
| West Bengal | 14.9 | 14.3 | 4.2 | 56.5 | 63.8 | 63.8 |
| Odisha | 14.3 | 15.7 | 3.2 | 29.2 | 22.6 | 32.5 |
| **India** | **11.7** | **11.3** | **2.6** | **59.6** | **63.2** | **61.7** |
| Source: Authors’ computation based on NSS data  **MPCE=**Monthly Per Capita Expenditure | | | | | | |
