# Supplementary material for: Unmet need for treatment-seeking from public health facilities in India: An analysis of sociodemographic, regional and disease-wise variations
Source: PLOS Glob Public Health. 2022 Apr 19;2(4):e0000148. doi: 10.1371/journal.pgph.0000148 (PMC10022036; doi:10.1371/journal.pgph.0000148)
Supplement: S4 Table — (DOCX) [file pgph.0000148.s004.docx]

**S4 Table. States wise variation for unmet need for treatment seeking from any public health facilities among those who have not taken treatment from the any public health facility by major diseases conditions in India, NSS 2004-2018. India**

| **States/Union Territory** | **Communicable Diseases** | | | **Non-Communicable Diseases** | | | **Others Diseases** | | |
| --- | --- | --- | --- | --- | --- | --- | --- | --- | --- |
|  | **NSS-2004** | **NSS-2014** | **NSS-2018** | **NSS 2004** | **NSS-2014** | **NSS-2018** | **NSS 2004** | **NSS-2014** | **NSS-2018** |
| Andaman and Nicobar Islands | 0 | 54.8 | 24.9 | 40.2 | 36.2 | 74.8 | 59.8 | 9 | 0.3 |
| Andhra Pradesh | 21.6 | 19.2 | 18.9 | 48.5 | 75.4 | 77.4 | 29.9 | 5.4 | 3.7 |
| Arunachal Pradesh | 78.5 | 82.6 | 70.5 | 11.5 | 17.5 | 29.5 | 10 | 0 | 0 |
| Assam | 50.6 | 16.6 | 55.2 | 28.9 | 52.5 | 42.6 | 20.5 | 30.9 | 2.2 |
| Bihar | 52.1 | 46.8 | 61 | 22.8 | 49.1 | 35.4 | 25.1 | 4.1 | 3.6 |
| Chandigarh | 33.9 | 50.7 | 21.3 | 24.2 | 36.6 | 68.1 | 41.9 | 12.7 | 10.6 |
| Chhattisgarh | 54.4 | 65.7 | 64.7 | 17.2 | 33.5 | 30.4 | 28.4 | 0.8 | 4.8 |
| Dadra and Nagar Haveli | 48.8 | 22 | 93.4 | 20.5 | 55.8 | 4.2 | 30.7 | 22.3 | 2.4 |
| Daman and Diu | 33 | 16.6 | 4.5 | 21.2 | 74.1 | 91.7 | 45.8 | 9.3 | 3.8 |
| Goa | 9.9 | 50.4 | 9.9 | 50.4 | 49.1 | 90.1 | 39.7 | 0.6 | 0 |
| Gujarat | 36.7 | 39 | 42.1 | 40.4 | 57.9 | 56.3 | 22.9 | 3.1 | 1.6 |
| Haryana | 30 | 55.8 | 55.1 | 34.7 | 41.4 | 42.1 | 35.3 | 2.8 | 2.8 |
| Himachal Pradesh | 28.4 | 34.1 | 49.2 | 46.7 | 63.1 | 43.5 | 25 | 2.8 | 7.3 |
| Jammu and Kashmir | 36.8 | 42.4 | 64.4 | 26.1 | 53.5 | 33.6 | 37.2 | 4.1 | 2 |
| Jharkhand | 47.7 | 41.3 | 61.1 | 19.5 | 48.4 | 35.6 | 32.8 | 10.4 | 3.3 |
| Karnataka | 28 | 39.3 | 34 | 44.5 | 57.8 | 63.7 | 27.5 | 2.9 | 2.4 |
| Kerala | 18.9 | 22.5 | 20.2 | 47.6 | 67.4 | 73.8 | 33.6 | 10.1 | 6 |
| Lakshadweep | 22.7 | 27.7 | 4.1 | 31.3 | 58.2 | 91.2 | 46.1 | 14.1 | 4.7 |
| Madhya Pradesh | 49.4 | 55.1 | 51.6 | 25 | 40.9 | 46 | 25.7 | 4.1 | 2.4 |
| Maharashtra | 28.5 | 47.7 | 41.7 | 41.4 | 48.4 | 54.4 | 30 | 3.9 | 3.9 |
| Manipur | 39.9 | 57.9 | 64.5 | 26.1 | 28.8 | 16.5 | 34 | 13.3 | 19 |
| Meghalaya | 54.7 | 91.6 | 92 | 7.2 | 8.4 | 8 | 38.1 | 0 | 0 |
| Mizoram | 45.9 | 9.5 | 21 | 34.9 | 89.6 | 73.9 | 19.2 | 0.9 | 5.1 |
| Nagaland | 94.1 | 87.4 | 20.4 | 1.3 | 11.5 | 77.3 | 4.6 | 1.2 | 2.4 |
| Delhi | 28.6 | 65.3 | 71.8 | 38.7 | 33.9 | 28.2 | 32.7 | 0.8 | 0 |
| Puducherry | 9.1 | 41.3 | 34.7 | 63.9 | 55 | 59.8 | 27 | 3.7 | 5.5 |
| Punjab | 27.9 | 41.6 | 48.3 | 39.9 | 52.4 | 50.1 | 32.2 | 6 | 1.6 |
| Rajasthan | 39.9 | 48.7 | 46.8 | 30.1 | 46.1 | 47.5 | 30 | 5.2 | 5.8 |
| Sikkim | 56.7 | 7.6 | 42.8 | 20.5 | 77 | 57.2 | 22.9 | 15.4 | 0 |
| Tamil Nadu | 27.6 | 29.4 | 33.9 | 39.7 | 67.1 | 63.8 | 32.7 | 3.5 | 2.4 |
| Telangana | 21.6 | 42.5 | 34.1 | 48.5 | 52.1 | 57 | 29.9 | 5.5 | 8.9 |
| Tripura | 41.2 | 59.7 | 40.3 | 22 | 28.2 | 47.7 | 36.8 | 12.2 | 12 |
| Uttar Pradesh | 46.9 | 50 | 62.8 | 25.3 | 42.6 | 31.5 | 27.9 | 7.4 | 5.7 |
| Uttarakhand | 47.2 | 56.1 | 59.3 | 29.3 | 38.5 | 32.5 | 23.5 | 5.4 | 8.2 |
| West Bengal | 21.2 | 31.9 | 28.4 | 39.6 | 56.5 | 66.8 | 39.2 | 11.6 | 4.8 |
| Odisha | 46.3 | 44.9 | 41.6 | 18.7 | 53.3 | 54.7 | 35.1 | 1.8 | 3.7 |
| **India** | **33.7** | **37.7** | **41.5** | **35.9** | **56.0** | **54.2** | **30.4** | **6.3** | **4.3** |
| Source: Authors’ computation based on NSS data | | | | | | | | | |
