# Supplementary material for: Unmet need for treatment-seeking from public health facilities in India: An analysis of sociodemographic, regional and disease-wise variations
Source: PLOS Glob Public Health. 2022 Apr 19;2(4):e0000148. doi: 10.1371/journal.pgph.0000148 (PMC10022036; doi:10.1371/journal.pgph.0000148)
Supplement: S5 Table — (DOCX) [file pgph.0000148.s005.docx]

**S5 Table. States wise variation for unmet need for treatment seeking from any sources among those who have not seeking treatment from any sources by major diseases conditions in India, NSS 2004-2018. India**

| **States/Union Territory** | **Communicable Diseases** | | | **Non-Communicable Diseases** | | | **Others Diseases** | | |
| --- | --- | --- | --- | --- | --- | --- | --- | --- | --- |
|  | **NSS-2004** | **NSS-2014** | **NSS-2018** | **NSS 2004** | **NSS-2014** | **NSS-2018** | **NSS 2004** | **NSS-2014** | **NSS-2018** |
| Andaman and Nicobar Islands | 0 | 97.5 | 0 | 87.7 | 2.5 | 100 | 12.3 | 0 | 0 |
| Andhra Pradesh | 22.1 | 30.6 | 1.5 | 31.7 | 63.1 | 72.1 | 46.2 | 6.3 | 26.4 |
| Arunachal Pradesh | 88.6 | 65.2 | 69.6 | 11.4 | 34.8 | 30.5 | 0 | 0 | 0 |
| Assam | 53.7 | 70.8 | 55.8 | 15.9 | 29.2 | 44.2 | 30.5 | 0 | 0 |
| Bihar | 36.9 | 76.2 | 71.3 | 30.8 | 23.8 | 27 | 32.3 | 0 | 1.7 |
| Chandigarh | 0 |  | 46.5 | 1.1 |  | 26.7 | 98.9 |  | 26.7 |
| Chhattisgarh | 1 | 68.5 | 80.1 | 30.7 | 31.4 | 19.7 | 68.4 | 0.1 | 0.2 |
| Dadra and Nagar Haveli | 0 | 52.6 |  | 100 | 47.4 |  | 0 | 0 |  |
| Daman and Diu | 0 | 89 |  | 81.4 | 0 |  | 18.6 | 11 |  |
| Goa | 0 | 95.9 | 100 | 89.5 | 4.1 | 0 | 10.5 | 0 | 0 |
| Gujarat | 24.1 | 56.1 | 67.3 | 40.8 | 43.8 | 32.6 | 35.1 | 0.1 | 0.1 |
| Haryana | 22 | 35.4 | 11.2 | 57.9 | 64.1 | 84.6 | 20.2 | 0.5 | 4.2 |
| Himachal Pradesh | 35.9 | 63.2 | 34.9 | 21.8 | 29.1 | 65.1 | 42.3 | 7.8 | 0 |
| Jammu and Kashmir | 10.8 | 69.5 | 68.2 | 64 | 29.6 | 31.8 | 25.3 | 1 | 0 |
| Jharkhand | 94.7 | 48 | 59.5 | 0 | 41.6 | 40.3 | 5.3 | 10.4 | 0.2 |
| Karnataka | 12.1 | 58.1 | 70 | 53.2 | 41.3 | 30 | 34.7 | 0.6 | 0 |
| Kerala | 23.2 | 45.6 | 20.4 | 20.4 | 46.6 | 65.1 | 56.4 | 7.8 | 14.6 |
| Lakshadweep | 6.6 | 50.5 |  | 24.4 | 18.8 |  | 69 | 30.7 |  |
| Madhya Pradesh | 28.6 | 54.3 | 37.9 | 18.9 | 43.7 | 62.1 | 52.5 | 2 | 0 |
| Maharashtra | 10.5 | 62.7 | 60.3 | 31.5 | 27.9 | 27.6 | 58 | 9.4 | 12.1 |
| Manipur | 73.7 | 91 | 41.7 | 13.2 | 8.1 | 58.4 | 13.2 | 0.8 | 0 |
| Meghalaya | 66.8 | 53.1 |  | 12.2 | 39.9 |  | 21 | 7 |  |
| Mizoram | 72 | 63.5 | 38.2 | 15.5 | 34.3 | 45.7 | 12.5 | 2.2 | 16.2 |
| Nagaland |  | 83.9 | 92 |  | 16.1 | 5.6 |  | 0 | 2.3 |
| Delhi | 0 | 55.1 | 99.3 | 100 | 22.4 | 0.7 | 0 | 22.4 | 0 |
| Puducherry | 8.5 | 45.1 | 100 | 12.1 | 54.5 | 0 | 79.4 | 0.4 | 0 |
| Punjab | 6 | 59 | 27.7 | 43.9 | 32 | 66.2 | 50.1 | 9.1 | 6.1 |
| Rajasthan | 42.3 | 84.9 | 66.5 | 16.8 | 12.2 | 29.5 | 40.9 | 2.9 | 4.1 |
| Sikkim | 36.3 | 27.6 | 16.7 | 7.3 | 60.6 | 83.3 | 56.4 | 11.8 | 0 |
| Tamil Nadu | 29.1 | 56.7 | 16.7 | 34.2 | 40.6 | 83.3 | 36.7 | 2.7 | 0 |
| Telangana |  | 31.7 | 76.3 |  | 68.4 | 17.8 |  | 0 | 5.9 |
| Tripura | 34.2 | 74.3 | 100 | 10.5 | 25.7 | 0 | 55.3 | 0 | 0 |
| Uttar Pradesh | 41.6 | 51.2 | 53.7 | 20.1 | 41.5 | 40.1 | 38.3 | 7.3 | 6.2 |
| Uttarakhand | 3.1 | 72.6 | 73.9 | 11.8 | 27.4 | 26.1 | 85.1 | 0 | 0 |
| West Bengal | 30.1 | 57.4 | 11.3 | 29.9 | 41 | 84.8 | 40 | 1.6 | 3.9 |
| Odisha | 52.7 | 67.5 | 67.6 | 11.5 | 26.8 | 23 | 35.7 | 5.8 | 9.4 |
| **India** | **30.54** | **57.6** | **44.1** | **27.2** | **38.4** | **50.1** | **42.3** | **4.0** | **5.82** |
| Source: Authors’ computation based on NSS data | | | | | | | | | |
