# Supplementary material for: Unmet need for treatment-seeking from public health facilities in India: An analysis of sociodemographic, regional and disease-wise variations
Source: PLOS Glob Public Health. 2022 Apr 19;2(4):e0000148. doi: 10.1371/journal.pgph.0000148 (PMC10022036; doi:10.1371/journal.pgph.0000148)
Supplement: Attachment — Submitted filename: Response to Reviewers_PGPH-D-21-00530.docx [file pgph.0000148.s006.docx]

**Author’s response to the Reviewer’s comments**

Please find the comments and their respective responses below

**Reviewer’s #1 comments and Author’s responses:**

**Comments:** The authors tried to address the research gaps in unmet need for treatment-seeking and medical consultation, especially for public health facilities across sociodemographic characteristics, regions, and specific diseases. However, there are some statistical concerns in this manuscript especially in the methodology, analytical approach and reporting of finding and are described below.

**Response:** We wish to thank the reviewer for motivating feedback. The authors are grateful to the esteemed reviewer for appreciating the relevance of our work. We have considered all the suggestions given by reviewer # 1 in the revised version.

Our detailed reply to each and every point raised by the Reviewer’s #1 is given below:

**Query 1:** In order to address it, authors used data from three rounds of the National Sample Survey (2004, 2014, 2018). However, the rationale for performing such comparing the 3 timepoint data analysis is missing, although the population structure, sampling method or sample size differs.

**Response:** Thanks to the reviewer for this important observation. The idea about three-time period was to explore the unmet need for public health facilities over the period and try to see whether different policy initiatives taken by the governments have improved the treatment seeking from public sources or not. We have mentioned a few names such as the National Rural Health Mission launched in 2005, the Universal Health Coverage adopted in 2012 and the National Health Mission started in 2013. The National Sample Survey (NSS) is a cross-sectional national level representative survey following the same sampling method across different rounds. The survey of design and sample weights have been devised in a such a way that it is not affected by the population structure, sampling strategy or sample size if one compares estimates over the different rounds. In fact, one of the objectives of carrying the NSS on social consumption is to periodically assess the progress of the current policy initiatives and future policy implications. Several important studies in the past have been published in making such attempts. We have also made the necessary changes in the revised version of the paper.

**Query 2:** In the abstract as well as in main manuscript authors mentioned about “multivariate” analysis. None of the methods adopted in the manuscript is multivariate. It may be corrected as “multivariable analysis”.

**Response:** Thanks for your observation. We have made the necessary changes in the revised version of the paper.

**Query 3:** In the “Data and Methods” section, the authors specified that a cross-sectional secondary data from three waves of the National Sample Survey. Whether the sampling strategy remained same for all 3 waves?

**Response:** Thanks for your observation. NSS uses the same sampling strategy of multistage sampling for all three rounds. In the first stage of sampling, villages/urban blocks are selected using the probability proportional to size (PPS) sampling, while in the second stage, households are selected using the systematic random sampling from the selected villages/urban blocks. However, the authors taken care of research deign adjustment by weighed analysis in all three rounds and it is mention in the paper in the Statistical Analysis Sub-section.

This study used the ArcGIS 10.7.1 (ESRI 2020 (10.7.1)) and the STATA (version 13) statistical analysis software for all the analyses (Stata Corp., 2013) after adjusting the survey design and the sampling weight with the SVY command (Korn EL & Graubard BI, 1990).

**Query 4:** Page 10, Para 2: Statistical analysis section: Needs to be polished. Authors specified that “In the first part of the analysis, descriptive analysis …….., while in the second part, bivariate estimates … and In the third part of the analysis, …” Instead authors may be instructed to specify the statistic used and test of significance used for bivariate analysis and so on.

**Response:** Thanks again for this important observation on statistical analysis section. Now this section is revised as per your suggestions.

**Query 5:** Page 10, Para 2: Further multinomial logit regression is not a multivariate analysis. It may be modified as multivariable analysis.

**Response:** Thanks for your observation. We have made the necessary changes in the revised version of the paper.

**Query 6:** Page 10, Para 2: Moreover, the equation for multinomial logistic regression specified is not a general form and is a special case with only two independent variables. This may be replaced with a general form of ‘n’ independent variables

**Response:** Thanks for your observation. We have made the necessary changes in the revised version of the paper.

**Query 7:** Page 10, Last Para: The author mentioned that the analysis was carried out after adjusting the survey design and the sampling weight. Are the weight used same for all the waves or for each wave separate weight computation was done and considered for this analysis? Mention about this in the manuscript for more clarity.

**Response:** Thanks for your observation. We have made the necessary corrections to make the things clearer on this aspect in the revised version of the manuscript.

**Query 8:** Page 11, Table 1: Characteristics described in the table include various socio-demographic characteristics. Hence socio-economic characteristics may be replaced with socio-demographic characteristics and should reflect where ever applicable. Moreover, the sequence of the information differs from the standard representation. Specify the n first followed by the % information. Further, as the sample size varies between the waves, it’s good to provide those number under each columns of NSS-2004, NSS-2014 & NSS-2018.

**Response:** Thanks for your suggestion. We have made the necessary changes and replace socio-economic characteristics with socio-demographic characteristics. We also made the n first followed by the % in the respective places and provided n in column NSS-2004, NSS-2014 & NSS-2018 in the revised version of the manuscript.

**Query 9:** In the titles of Table 1 to 5 mentioned “NSS, 2014-2018”, though 2004 summary were mentioned. This needs to be corrected.

**Response:** Thanks for your observation. We have made the necessary changes in Table 1 to 5 as 2004 instead of 2014 in the revised version of the paper.

**Query 10:** Page 15, Table 3: Provide the description of notations and abbreviations used as footnotes.

**Response:** Thanks for your observation. We have made notations and abbreviations used as footnotes in tables in the revised version of the paper.

**Query 11:** Page 16, Table 5: Provide the description of notations and abbreviations used as footnotes

**Response:** Thanks for your observation. We have made notations and abbreviations used as footnotes in tables in the revised version of the paper.

**Query 12:** Page 19, Table 7: There is some typo error in the title and may be corrected

**Response:** Thanks for your observation. We have corrected the typo error made in the title of the Table 7

**Query 13:** Number of tables found too many, some of these tables may be kept in the manuscript as supplementary files.

**Response:** Thanks for your feedback. We will surely do the same as per the journal’s policy in the revised version of the paper. However, these many the minimum number of tables are necessary to provide a sequential and logical understanding of the issue dealt in this paper.

**Query 14:** Are there any information on Unmet need for treatment-seeking available in NFHS-4 finding. In that case a comparison of these estimates with respect to the findings of NFHS may be described in discussion section.

**Response:** Thanks for your feedback. NFHS focuses only on a few aspects/diseases of treatment seeking among children (<5 year) and women (15-49) and men (15-49). Therefore, a comparison of our findings with NFHS-4 may mislead to a reader. Unmet need for family planning methods in all the round of NFHS is a unique indicator which is usually devised based on women’s reproductive status and desire of the next child indicating what proportion of women were not using any method of family planning though they require to use a method to achieve their fertility goals. This paper does not deal with any of such conditional indicator by age or health condition or use of health facility seeking any family planning method. Hence, we preferred not to make such comparisons in the revised manuscript. However, as per your suggestion, we have mentioned NFHS findings regarding treatment not sought for diabetes, Asthma, Goiter/any thyroid and any hearth disease among women and men (15-49) at the national level in the discussion section of the paper.

**Query 15:** The pattern of trend seen on Unmet need for treatment seeking as well as medical consultation across the different rounds may be discussed.

**Response:** Thanks for your feedback on this aspect. We have made them in the revised version of the manuscript.

**Reviewer’s #2 comments and Authors’ responses:**

**Comment:** The article talks about important topic unmet need of medical consultation in government health care system.

**Response:** We wish to thank the reviewer for important observations and feedback. The authors are grateful to the esteemed reviewer for appreciating the relevance of our work. We have considered all the suggestions given by reviewer# 2 in the revised version.

Our detailed reply to each and every point raised by the Reviewer’s #2 is given below:

**Comment 1:** The article is written well but for Indian audience. For international reader it has to elaborate in details every terminology used. The manuscript is not written clearly enough to be accessible to non-specialists.

**Response:** Thanks for this observation. We have made progress on this aspect and clarified different terms in the revised version. However, certain technical terms cannot be replaced as they standard term in the literature of statistical analysis and we afraid of replacing them may create further confusions to the potential readers.

**Comment 2:** The study is secondary data analysis up to year 2018. During COVID Pandemic the health care situation across the country has dramatically changed. Can this study results generalized in current situation? Explain

**Response:** Thanks for your important observation. The revised version of the paper has mentioned the rational of the study clearly which we could not do in the first version. This paper would like to see whether past policy initiatives have made any significant improvement in reducing unmet need in treatment seeking from public health facilities. The Covid-19 pandemic is an unprecedent event since India’s public health system came into existence. We do understand there might be many who could not seek service from the public health system as most of public health facilities were fully absorbed in treating Covid-19 patients. However, the situation has completely improved now and the system is again on its usual track. Your suggestion is really thought provoking and a separate study with required latest data is very much desirable. Some studied have already been conducted but these are sporadic and are not nationally representative. Our study deals with the situation of unmet need for treatment seeking for public health facilities in normal circumstances.

Title

**Comment 3:** Title is “Unmet need for treatment-seeking” The article mentions more of unmet need of medical consultation. Need to be revise

**Response:** Thanks again for your feedback on the title. In this manuscript, we are primarily dealing with unmet need for treatment-seeking for public health facilities. However, we have used “medical consultation” and “treatment-seeking interchangeably”. We have mentioned this in the method section in the revived version of the manuscript. We would like to retain “treatment-seeking” due to the reason that public health facilities in India do not provide “medical consultation” alone as per protocol. Either they treat or refer to a patient to higher level of facilities if any reason treatment could not be given. In rarest of rare circumstances/in emergency when patients could not be treated at a particular level of facilities or not referred to a higher-level facility, they might be given medical prescription or medical consultation for seeking treatment from other sources including private and others (profit or non-profit). However, NSS considered unmet need for treatment seeking from any sources and from public health facilities. Thus, in the revised version we have made it much clearer it in the operational definition of variables in the output variable sub-section.

Abstract
**Comment 4:** “Especially” in abstract: Manuscript does not give details for private health facilities” need to remove this word

**Response:** Thanks for your observation. We have removed “Especially” from in the revised version of the paper.

Introduction

**Comment 5:** It is written taking public health facilities as a single unit. Assessing Government Medical college, district hospital down to subcentre in single note is lacking clarity for the Indian Health Care System. Need to revised by level of health care for a clear view of international readers.

**Response:** Thanks for your valuable suggestion. We have added a para on this aspect in the Data and Method section of the revised version of manuscript.

**Comment 6:** It elaborated only the lacunas and challenges from the health care facility point. Treatment seeking is missing and need to added.

**Response:** Thanks for your comment. We have taken into consideration this in the revised manuscript.

**Comment 7:** The term “unmet need” to be standardize across the article. It is bemusing.

**Response:** Thanks for your feedback. This has been done in the revised version of the manuscript.

**Comment:** 8. In title, abstract and result it mentions Unmet need for treatment-seeking, unmet need of medical consultation

**Response:** Thanks for your valuable comment. There are only two types of unmet need which has been covered in this study which are unmet need for treatment seeking from any sources and unmet need for treatment seeking from public health facilities. Where unmet need for treatment seeking is general term it means it covers unmet need from any kind of health care whereas unmet need for treatment seeking from public health facility is specific unmet need for utilising public healthcare facilities. Now changes have been made to the manuscript and these terms have been made uniform throughout the manuscript.

**Comment:** 9. In methodology operational definition are for unmet need for public health facility and unmet need for health care

**Response:** Thanks for your valuable comment. The response of this comment has been covered while preparing the response of comment number 8.

**Comment:** 10. In methodology it also mentions unmet need for medical treatment advised

**Response:** Thanks for your valuable comment. The response of this comment has been covered while preparing the response of comment number 8.

**Comment:** 11. Provide operational definition for Unmet need for treatment-seeking, unmet need of medical consultation, unmet need for medical treatment advised as they same as unmet need for public health facility and unmet need for health care need to be clarified.

**Response:** Thanks for your valuable comment. The response of this comment has been covered while preparing the response of comment number 8.

**Comment:** 12. Details of the methodology is not sufficient to allow to be reproduced

**Response:** Thanks for your feedback. We have revised the methodology section as per your suggestion.

**Comment:** 13. Methodology of NSS Survey is written, please revise the methodology to the present study methodology: how the data was retrieved, how were the variable chosen, how much is missing data etc, May be a flowchart

**Response:** Thanks for your feedback. We have added a flowchart for better understanding in the method section.

**Comment:** 14. Mention what is MPCE Quintile

**Response:** Monthly per capita expenditure (MPCE) is used as a proxy for the economic status of the household, due to absence of income data. Further MPCE is categorised in five quintiles namely poorest, poorer, middle, richer and richest.

**Comment:** 15. Mention what is SC/ST and OBC

**Response:** Thank you for drawing attention to elaborate the terms in the text. We have explained them in detail. In NSS data caste categories are classified as Scheduled Caste (SC), Scheduled Tribe (ST), Other backward class (OBC) and Others. In our study we merged first two categories, SC and ST to one category as SC/ST. Caste/tribe has significant relevance in the Indian context for any social and health indicator. Under the varna system, humans were divided into four classes. The bottommost rung of this system includes 'scheduled castes/tribes,' who were allowed to do derogatory and scavenging work only. People from these strata lack access to information and health services while the 'other backward classes’ (OBC) comprise those deprived of health services primarily due to social and economic constraints. The 'other' category comprises those not comprised in any of the above categories.

Now it is mentioned in the Table S2, description and Scale of measurements of explanatory variables in the revised manuscript.

**Comment:** 16. Mention about Indian Geography to make readers understand what is North, West, East, Northeast, South, central

**Response:** India was divided into six regions based on geographical location and cultural settings. The six regions consist of North (Jammu and Kashmir, Himachal Pradesh, Punjab, Haryana, Rajasthan, Delhi and Uttaranchal), Central (Uttar Pradesh, Madhya Pradesh and Chhattisgarh), East (Bihar, Jharkhand, West Bengal and Orissa), North-East (Arunachal Pradesh, Assam, Manipur, Meghalaya, Mizoram, Nagaland and Tripura), West (Gujarat, Maharashtra and Goa), and South (Andhra Pradesh, Karnataka, Kerala and Tamil Nadu).

Now it is mentioned in the Table S2, description and Scale of measurements of explanatory variables in the revised manuscript.

Results
**Comment:** 17. Authors mentioned “The unmet need for medical consultation at public health facilities remained high at 60% in 2004 to 62% in 2018” Can provide stratification.

**Response:** Thanks for your feedback. We have done stratification as per three levels of facilities (primary, secondary and tertiary level).

In India, the public health system is called three-tyre health system. At the primary care level, the minor ailment and maternal and child health related services are provided though sub-health centre (SHC) and primary health centre (PHC). The secondary health care level includes district hospitals (DH) and community Health Centre (CHC) at block level. Tertiary Health care refers to a third level of health system, in which specialized consultative care is provided usually on referral from primary and secondary medical care. This includes medical colleges, large district hospitals or research hospitals. We have mentioned service offers and treatment given at each level in the data and method section.

**Comment:** 18. Result is presented monotonously in table format. Table 2 to be in single Map

**Response:** Thanks for your valuable comment. Now table 2 is moved in supplementary appendix as table S3

**Comment:** 19. Table 4 Morbidities to be coded in standard format like ICD or SNOMED CT

**Response:** Thanks for your valuable comment. It is based on the ICD-10 classification.

http://www.who.int/classifications/apps/icd/icd10online, 2007

Discussion
**Comment:** 20. The data and analyses do not fully support the conclusion

**Response:** Thanks for your valuable comment. Appropriate changes have been made to revised manuscript which are mentioned in the responses of comments given below.

**Comment:** 21. It mentions “One of the important findings of this study is that the main reason for not seeking medical treatment was the ailment not being considered serious enough”. Data description for not seeking treatment is missing in result section.

**Response:** The table and results has been added in the result section regarding reason for not seeking medical treatment in the revised version of the manuscript.

**Comment:** 22. It mentions “This study also shows that the utilization of private healthcare facilities is more common among the elderly population (60+).” Data description private health facility or proportion of government health facility care available is missing in results

**Response:** This part of the discussion has been removed in the revised manuscript because this result does not look fit for the objectives of this study in the revised version of the manuscript.

Conclusion

**Comment:** 23. It mentions “Universal Health Coverage”

**Response:** Thanks for your feedback. We have mentioned in the revised version of the manuscript.

**Comment:** 24. Compare results with universal health coverage issues in discussion

**Response:** Thanks for your feedback. Done in the revised version of the manuscript.

**Comment:** 25. The discussion, result and conclusion need to be synchronized

**Response:** Thanks for this particular suggestion. We have revised these section accordingly in the revised paper.

Minor Changes

**Comment:** 26. Page 4 Key Massages replace to Key Messages

**Response:** Thanks for your important observation. We made a correction in the revised version of the paper.

**Comment:** 27. Keywords to be as MeSH PubMed

**Response:** Thanks for your feedback. We have streamlined keyword that can be suited to MeSH PubMed search.

**Comment:** 28. Table 3 title font size “sociodemographic”

**Response:** Thanks for your important observation. We made a correction in the revised version of the paper.

**Comment:** 29. In result tables; Non-SC/ST/OBC replace to “unreserved or general”

**Response:** Thanks for your important observation. We made a correction in the revised version of the paper. Unreserved is better suited for employment purpose but ‘others’ and ‘general’ are frequently used in the academic context.

**Comment:** 30. The study itself show sufficient potential that the authors should be encouraged to resubmit a revised version

**Response:** We wish to thank the reviewer for motivating feedback. The authors are grateful to the esteemed reviewer for appreciating the relevance of our work. We have considered all the suggestions given by you and other reviewers and we are submitting the revised version of the manuscript for possible publication**.**

**Comment:** 31. Original data of NSS is used link presented

**Response:** All the NSS rounds data are in public domain and available to individuals on <http://www.mospi.gov.in/>

**Reviewer’s #3 comments and Authors’ responses:**

**Comment:** The authors have addressed have a very important topic. Health care seeking and addressing unmet needs of the population in the long run is very crucial for policy makers to achieve universal health coverage in the country. I have few suggestions and comments.

**Response:** We wish to thank the reviewer for important observations and feedback. The authors are grateful to the esteemed reviewer for appreciating the relevance of our work. We have considered all the suggestions given by reviewer# 2 in the revised version.

Our detailed reply to each and every point raised by the Reviewer’s #3 is given below:

**Comment 1:** A separate annexure may be provided to mention which block of NSSO data was used and how the data was merged. I am concerned whether the variables/questions remained the same across the 3 rounds and the answers/choices provided by the respondents had the same coding.

**Response:** variables/questions remained the same across the 3 rounds and the answers/choices provided by the respondents had the same coding.

**Comment 2:** More details of multi-collinearity screening need to be added. May be in the annexure.

**Response:** Thanks, we have provided the more information on this aspect.

**Comment 3:** A chi-square for trend can be added in Table 1.

**Response:** Table 1 provides frequency distribution of the sample by background characteristics. The figures are not the prevalence/rate.

**Comment 4:** Can Table 2 be shown as a figure across 6 regions (separately for treatment seeking and consultation from public facility)?. The table appears too crowded. On seeing a trend figure, I am sure more interesting points can come out.

**Response:** Thanks for your comment we agree it is too crowded. However, we thing information kept in the table are necessary for program managers.

**Comment 5:** Elaborate and provide the full form of IHDS-II. Is this similar to NSSO? Can be mentioned in the methods too.

**Response:** Thanks for your important observation. It is typing mistake. It is NSS survey. We made a correction in the revised version of the paper.

**Comment 6:** In Table 3 can we have the ‘p’ values. That will be more useful. Remove the “***”

**Response:** Thanks for your important observation. In our experience, sometimes general readers find more convenient to understand the level of significance at a given level shown by ‘*’ rather than exact value of ‘p’. However, if you still insist we can generate and place ‘p’ values in the approved version.

**Comment 7:** Why Table 4 doesn’t have a Chi-square done with a ‘p’ value? I would like to see the change over survey rounds and not within rounds. This applies to all tables.

**Response:** Thanks for your observation. However, we have not tested chi-square p-value for time trend in our analysis.

**Comment 8:** The *, # and ns can be removed in Table 5. The confidence intervals are more than enough to interpret whether the aOR was significant or not.

**Response:** Thanks for your important observation. We would like to maintain the uniformity in presenting results across tables. Therefore, we think that ‘*’ (at 0.05 of level of significance) and 95% confidence interval are useful general readers, and the same has been followed in the table 2.

**Comment 9:** Table 6. I recommend for uniformity; it can be aligned similar to Table 3 & Table 4. Take Quality and show the values for 2004, 2014 and 2018 side by side. Take waiting and show the values of 2004, 2014 and 2018 side by side. Show the chi-square for trend ‘p’ value.

**Response:** Thanks for your observation. We have done the changes in table as per your suggestion. However, we have not tested chi-square p-value for time trend.

**Comment 10:** Table 7. Same as above

**Response:** Thanks for your observation. We have done the changes in table as per your suggestion. However, we have not tested chi-square p-value for time trend in our analysis.

**Comment 11:** Consent for publication needs to be changed to PloS Global health

**Response:** Thanks for your observations and valuable suggestions. We have made corrections in the revised version of the manuscript.

**Comment 12:** In the author contribution, I noticed a RR. Are we missing anyone or is it RY?

**Response:** Thanks for your meticulous review. It is RY. We have made the correction in the revised version of the paper.
