# Supplementary material for: Unmet need for treatment-seeking from public health facilities in India: An analysis of sociodemographic, regional and disease-wise variations
Source: PLOS Glob Public Health. 2022 Apr 19;2(4):e0000148. doi: 10.1371/journal.pgph.0000148 (PMC10022036; doi:10.1371/journal.pgph.0000148)
Supplement: Attachment — Submitted filename: Response to Reviewers_PGPH-D-21-00530_R3.docx [file pgph.0000148.s007.docx]

**Author’s response to the Reviewer’s comments**

Please find the comments and their respective responses below.

**Reviewer’s #1 comments and Author’s responses:**

**Comments:** No Response. All comments have been addressed.

**Response:** The authors are grateful to the esteemed reviewer for appreciating the relevance of our work.

**Reviewer’s #2 comments and Authors’ responses:**

**Comment:** All comments have been addressed.

**Response:** We wish to thank the reviewer for important observations and feedback. The authors are grateful to the esteemed reviewer for appreciating the relevance of our work and satisfied by authors’ reply to the comments. We have considered all the suggestions given by reviewer# 2 in the revised version.

Our detailed reply to each and every point raised by the Reviewer’s #2 is given below.

**Comment 1:** Affiliation: PHD to Ph.D.

**Response:** Thanks for the observation. We have made the correction in Affiliation from PHD to Ph.D. in the revised version of the paper.

**Comment 2:** Abstract: Result- last line "unment" correct the spelling

**Response:** Thanks for this important observation. We have made the correct in the revised version of the manuscript.

**Comment 3:** Rationale: national rural health mission: Uppercase

**Response:** Thanks again for your feedback. We have capitalized the first letter of each word (National Rural Health Mission) in the revised version of the paper.

**Comment 4:** Outcome variables:

first para Second last line "logtistic and multinomial" correct the spelling

last line " The operational definitions of all the these outcomes variables are

given below" to be shifted to appropriate place

third para- first line "three-tyre health system" to three-tier

last word "thospitals" correct the spelling

Reason for not seeking treatment at all from any sources: "neibourhood" correct the spelling

Reasons for unmet need for treatment seeking any public health facilities:"categoried them into" correct the spelling

**Response:** Thanks for your observation. We have made all the above corrections in the revised version of the paper. We have placed under the title “A Brief Description of India’s Healthcare System Functioning” taking out from the **outcome variable**s sub-heading. It seems more suitable placing in the rationale of the study.

**Comment 5:** Statistical analysis:

"treatrment" correct the spelling

last para "three rounds sepretaly" correct the spelling.

**Response:** Thanks for your valuable suggestion. We have made the correction at the respective places in the revised version of the paper. We really appreciate the reviewer for your meticulously reading our paper.

**Comment 6:** Tables foot notes: Sheduled Caste/Sheduled Tribes correct the spelling

OBC=Others Backwords Classes, correct the spelling

**Response:** Thanks for your comment. We have made the corrections at the respective places in the tables footnotes in the revised version.

**Comment 7:** Figure 3 Caption " unmet" uppercase.

**Response:** Thanks for your important observation. We have made the correction in the revised version of the paper.

**Reviewer’s #3 comments and Authors’ responses:**

**Comment:** Reviewer #3: The authors have built the background to the analysis and the rationale very well this time around.

**Response:** The authors are grateful to the esteemed reviewer for appreciating the relevance of our work.
